# Effects of Biochar on the Availability of Trace Elements in Different Types of Soil

**DOI:** 10.3390/toxics13030169

**Published:** 2025-02-27

**Authors:** Shuaihui Ma, Shuai Ma, Weiqin Yin, Shengsen Wang, Haijun Sheng, Xiaozhi Wang

**Affiliations:** 1 College of Environmental Science and Engineering, Yangzhou University, Yangzhou 225127, China; shuaihuima@163.com (S.M.);; 2 Joint International Research Laboratory of Agriculture and Agri-Product Safety of Ministry of Education of China, Yangzhou University, Yangzhou 225127, China; 3 Jiangsu Collaborative Innovation Center for Solid Organic Waste Resource Utilization, Nanjing Agricultural University, Nanjing 210095, China

**Keywords:** biochar, red soil, yellow-brown soil, trace elements, availability, rice grains

## Abstract

To investigate the effects of biochar on the availability of trace elements (Fe, Mn, Cu, and Zn) in soils with different properties, biochar derived from wheat straw (WSBC) and peanut shells (PSBC) was added to red and yellow-brown soils for pot experiments. The results showed that WSBC and PSBC significantly increased the red and yellow-brown soils’ organic matter (SOM) and available potassium (AK), C, and C/N, especially with WSBC in red soil. The total and available amounts of trace elements in red and yellow-brown soil decreased after biochar was applied, where the effect of WSBC on the available of Fe, Mn, and Zn was greater than that of PSBC and the effect on the available contents of Fe, Mn, and Zn was less than that of PSBC. WSBC and PSBC decreased the contents of Fe, Mn, and Zn in the grains in both soils, while they increased the content of Cu in the grains. According to the results of a canonical correlation analysis, there was a competitive relationship between Mn and Cu in the grains. Fe and Zn in the grains were negatively correlated with AP in red soil and positively correlated with AP in yellow-brown soil. This study evaluates the effect of biochar on soil nutrient cycles, ultimately maximizing the application of biochar in the field of agriculture.

## 1. Introduction

Rice is a widely consumed staple food for more than half of the world’s population [1,2]. As the largest rice grain producer, China accounts for 28.9% of the world’s production, with 2119 million tons being produced in 2020 [3]. Trace elements such as iron (Fe), manganese (Mn), copper (Cu), and zinc (Zn) in soil play an essential role in the growth of rice. For example, Fe deficiency may result in leaf chlorosis, while Mn deficiency reduces tillering, Cu deficiency causes infertility and increased grain, and Zn deficiency causes seedlings to stiffen [4]. Trace elements with excessive amounts will also cause ion poisoning, dramatically affecting the yield of rice.

Trace elements are essential nutrients for plant growth and can only be absorbed and used by plants in their available state [5,6]. Although the total amount of trace elements in the soil is high, a lower effective content can also affect the growth and quality of plants. Recently, it has been reported that metal concentration in soils significantly decreased when biochar was added (Cd > Zn > Pb > Cu > Fe). Biochar application may promote the release of heavy metals and improve the bioavailability of heavy metals in soil. Furthermore, due to its high ash content, biochar can enhance the lime effect of soil, increasing soil pH, promoting ammonia volatilization, and reducing the bioavailability of trace elements such as Zn and Mn, which are necessary for crop growth. It was reported that biochar reduced the availability of Zn in soil and the content of Zn in rice and wheat grains [7]. The addition of biochar to alkaline calcareous soil hindered the adsorption of Fe by kiwifruit [8]. Nevertheless, there is still a lack of comprehensive understanding of the effects of biochar on the bioavailability of trace elements in soils.

As a renewable resource, biochar is often applied in soil for carbon fixation, soil improvement, and contaminated soil remediation. Previous studies have proved that the addition of biochar has achieved positive effects. For example, the existence of biochar can increase the accumulation of soil organic carbon and change the soil pH [9]. The acidity and alkalinity of soil are dominated by the concentrations of H^+^ and OH^−^ ions in the soil solution. Biochar contains alkaline substances, which can be quickly released after being added to acidic soil, neutralizing acidity and increasing pH levels in the soil. Moreover, biochar has a negative charge on its surface and a high cation exchange capacity, which can improve the adsorption capacity of soil for K^+^, Ca^2+^, and Mg^2+^, enhancing soil fertility and improving the plants’ environment. Biochar is an alkaline material, and the pH of red soil can be significantly increased through the application of biochar, effectively reducing the content of metal elements (i.e., Fe, Al, Cu, etc.) and increasing the bioavailability of the exchangeable base cations in the acidic soil. Biochar itself contains many nutrients that can be released into the soil. The loose and porous structures of biochar reduce the rate of water infiltration and enhance the ability to adsorb highly mobile and easily leached nutrients in the solution, which is beneficial for increasing the uptake of nutrients by plants. Therefore, biochar is considered an excellent material for improving soil [10].

The properties of biochar are influenced by the type of feedstock and pyrolysis temperature [11], and different types of biochar have different effects on soil and plant growth [12]. Therefore, the degree and mechanism of the influence of biochar on the bioavailability of trace elements in soils with different properties can be significantly different. In this study, we assume the following facts: (I) biochar can change the physical and chemical properties of soil, which can affect the bioavailability of trace elements. (II) The competition among trace elements results in significant differences in the contents of rice grains due to different soil and biochar properties. The results of this study will help to predict the quality of agricultural products in the soils of different regions and to evaluate the effect of biochar on soil nutrient cycling, ultimately maximizing the utilization of biochar in agriculture.

## 2. Materials and Methods

### 2.1. Characteristics of Soil and Biochar

In this experiment, two kinds of soil with obvious pH differences were used. Red soil was collected from Jian, Jiangxi Province, and yellow-brown soil was collected from Yangzhou, Jiangsu Province. Both of them were from the 0–20 cm topsoil layer. The physical and chemical properties of the two soils are illustrated in Table 1. The two soils were air-dried in 1 mm nylon for later use. Wheat straw (WS) and peanut shells (PSs) were used as raw materials; they were crushed to about 0.5 cm and then subjected to oxygen-limited pyrolysis at 500 °C for 2 h to obtain wheat straw biochar and peanut shell biochar, which were marked as WSBC and PSBC, respectively.

### 2.2. Experimental Design

A pot experiment with 10 kg of soil was carried out in a greenhouse, and the treatments were as follows: (I) red soil with no biochar was added as a control treatment (SCK), yellow-brown soil with no biochar treatment was added as a control treatment (CK); (II) red soil with 5% WSBC or PSBC was named SWS or SPS, while yellow-brown soil with 5% WSBC or PSBC was named WS or PS, respectively. (III) The entire growth period was managed by alternating dry and wet conditions, and each treatment was repeated three times. After the potting soil was flooded and stabilized for one week, Nanjing 46 rice seedlings with the same growth conditions were selected for transplanting. Three points were made in each pot, with three plants in each point. Conventional fertilization for rice production was consistently applied across all of the treatments.

The rice ripened at about 140 days, after which the grains were harvested and the rice rhizosphere soil was collected. The grains were dried in an oven at 80 °C to a constant weight and then crushed with a grinder through a 100-mesh sieve. The soil samples were screened after natural air drying and analyzed for their physical and chemical properties.

### 2.3. Biochar Property Analysis

The pH was measured with the pH–water (1:20 *w*/*v*) method by using a pH meter. Then, 1 g of biochar samples were weighed into a 30 mL ceramic crucible and burned to a constant weight; then, they were ashed in a muffle furnace at 800 °C for 4 h for ash content determination. The cation exchange capacity (CEC) was determined using a modified ammonium acetate displacement method related to the Gaskin method [13,14]. N, P, and K nutrients in biochar were digested with H_2_SO_4_-H_2_O_2_. N was determined using the Kjeldahl nitrogen method, P was measured using the molybdenum antimony colorimetric method [15], and K was determined via flame spectrophotometry (6400 A, Shanghai, China). In addition, the trace elements in biochar were determined via inductively coupled plasma mass spectrometry (ICP-MS) (i CAPQ, Thermo Fisher, Lenexa, KS, USA) using the dry-ashing HNO_3_ method.

The C and N in biochar were determined with an element analyzer (Vario EL cube, Elementar, Germany). Then, the C/N ratio was calculated. The specific surface area and pore size distribution were evaluated on a multifunctional adsorption instrument (Autosorb IQ3, Anton Paar, Ashland, Wilmington, DE, USA). The surface morphologies of the samples were inspected via field emission scanning electron microscopy (FESEM, S-4800, Hitachi, Tokyo, Japan). The functional groups were identified through micro-infrared spectroscopy (Cary 610/670, Varian, Palo Alto, Santa Clara, CA, USA).

### 2.4. Soil and Plant Property Analysis

Soil pH (the ratio of soil to water was 1:5) was measured with an acidity meter (PHS-3C, LeiCi, Shanghai, China). Soil organic matter (SOM) content was determined via the external heating of potassium dichromate and the titration method. Total nitrogen (TN) and total carbon (TC) in the soil were determined with an element analyzer (Vario EL cube, Elementar, Germany). Available phosphorus (AP) in acidic soil was extracted using NH_4_F-HCL, and AP in neutral and alkaline soil was extracted using 0.5 mol/L NaHCO_3_; both methods involved evaluation with a spectrophotometer (721, Shanghai, LeiCi). Available potassium (AK) was extracted with 1 mol/L CH_3_COONH_4_ and determined via flame photometry (FP640, Xinyi Instrument, Shanghai, China). The total amount of trace elements in the soil was digested with the HCl-HNO_3_-HF-HClO_4_ method. Available Fe, Mn, Cu, and Zn in the soil were extracted using a diethylenetriamine pentaacetic acid–calcium chloride–triethanolamine solution (soil: leaching solution = 1:4). Diluted hydrochloric acid was employed to adjust the pH of DTPA (0.005 mol/L)-CaCl_2_ (0.01 mol/L)-TEA (0.1 mol/L) extractant to 7.30. Trace elements in the grains were processed using the dry-ashing HNO_3_ method. All trace elements were determined via ICP-MS (i CAPQ, Thermo Fisher, USA).

### 2.5. Data Processing

The chemical properties of the soil were analyzed using SPSS 23. The data were analyzed using both descriptive and inferential statistics. Descriptive statistics (mean ± standard deviation) were used to summarize the data, while inferential statistics (ANOVA) were applied to assess the significance of differences between groups. Statistical significance was set at *p* < 0.05. The Origin Pro 2021 software was used for data processing.

## 3. Results and Discussion

### 3.1. Characterization of Biochar

The physicochemical characteristics of biochar derived from wheat straw and peanut shells through oxygen-limited pyrolysis at 500 °C are listed in Table 2. Both WSBC and PSBC showed a relatively high pH value ranging from 11.44 to 11.87. The ash contents of WSBC and PSBC were 28.99% and 39.67%, respectively. The higher ash content of biochar may have had a certain impact on the soil pH because ash usually contains alkaline substances, such as metal oxides. When biochar was added to the soil, these alkaline substances were released into the soil to neutralize the acids in it, which had a significant impact on the pH of the acidic soils. Alkaline soils already have a higher pH, so the addition of biochar will usually have a smaller effect on them. The nutrients, including N, P, Fe, Cu, and Zn, in PSBC are more plentiful than those in WSBC, and WSBC has greater values of K and C/N and a larger specific surface area, which is related to the properties of the raw materials.

**Figure 1:**
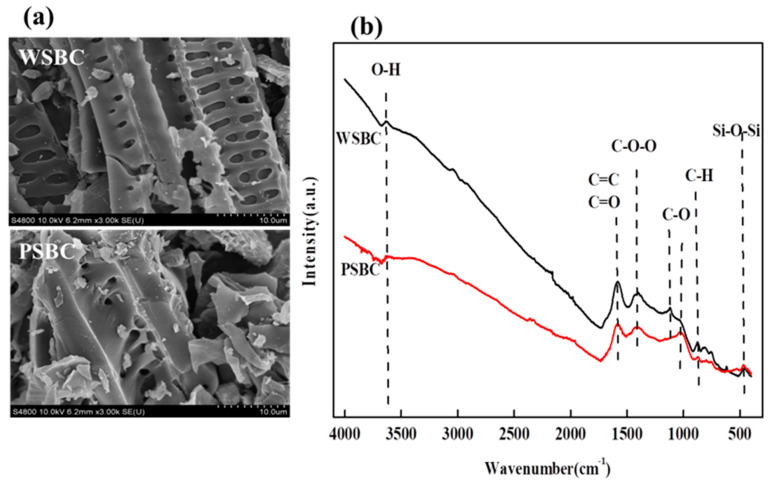
(**a**) SEM images and (**b**) FTIR spectra of WSBC and PSBC.

The SEM images shown in Figure 1a indicate that WSBC and PSBC are porous materials with irregular structures. Abundant pore structures caused by the templating facilitate the physical adsorption process between biochar and trace elements [16]. It was reported that the surface functional groups play an important role in the reaction between trace elements and biochar [17]. As demonstrated in the FTIR spectra displayed in Figure 1b, the broad peak located at around 3600 cm^−1^ due to the existence of O-H is identified in both samples. The biochar also displays a peak centered at 1629 cm^−1^ because of the stretching vibration of C=C and C=O bonds in aromatic carbons [18], and the intensity of the peak identified in PSBC decreases compared with that in WSBC, which may be due to the decomposition of chemical substances and the breaking of double bonds during the secondary pyrolysis of biomass [19]. In addition, peaks located at 550–1050 cm^−1^ can be ascribed to the vibration of C-H and C-O, and the characteristic peak intensity of WSBC is higher than that of PSBC, indicating that PSBC has more functional groups, mainly including olefins centered at 731 cm^−1^ and ethers identified at 1017–1098 cm^−1^ [20]. The peak centered at 473 cm^−1^ is attributed to the vibration of Si-O-Si in WSBC and PSBC due to the introduction of silica [21].

### 3.2. Effect of Biochar on the Chemical Properties of Soil

The pH, SOM, AP, AK, C, and N of the soil vary depending on the type of biochar added to different types of soil. The pH values of yellow-brown soil were higher than those of red soil (7.59–7.74 and 5.21–6.13) (Table 3). The pH increased by 22.96% and 17.68% after adding WSBC and PSBC into the red soil. Generally, the soil pH increased with the increase in the pH of biochar, but this was not observed in alkaline yellow-brown soils. SWS treatment possibly induced a high association of OH− and decreased H+ binding capacities at the exchange site, consequently increasing the pH of SWS [22]. This effect was greater in acidic soil, as the pH of acidic soil can be increased to a greater extent than alkaline soil [23]. Nevertheless, the difference in the pH of yellow-brown soil after the addition of biochar was not significant, which may have been because neutral yellow-brown soil has a good buffering performance, so the soil maintains a relatively stable pH.

Compared with the control group, the content of organic matter in the two kinds of soils treated with WSBC and PSBC increased by 673.87%, 278.93%, 89.73%, and 22.28%, respectively. The increase in SOM may be attributed to the content of organic carbon in the biochar (Table 2), which is consistent with the results provided by Karimi et al. [24]. The addition of WSBC and PSBC increased the AP by 182.67% and 3.50% in red soil, while the application of WSBC and PSBC increased it by 105.44% and 17.48% in yellow-brown soil, respectively, which was attributed to the high content phosphorus in PSBC. Other studies showed that the input of exogenous phosphorus will accelerate the mineralization of soil organic carbon by microorganisms and promote the degradation of organic carbon [25]. A significant difference between WSBC and PSBC was observed for the AK content in the two soils. It was increased with WSBC and PSBC by 1167.11% and 465.69% in the red soil and by 551.93% and 194.73% in the yellow-brown soil, respectively. The content of K in WSBC was about 5.16 times higher than that in PSBC. Therefore, there is a significant difference in the content of available K between the two soils treated with WSBC.

The application of biochar increases the total C, N, and C/N ratio, especially in red soil with poor organic matter. For total C, compared with the SCK treatment, the red soil treated with WSBC and PSBC increased by 663.01% and 267.12%, respectively. The difference in total N increased insignificantly, and the C/N ratio increased by 436.62% and 195.93%. In the yellow-brown soil, compared with the CK treatment, WSBC and PSBC increased the total C by 77.51% and 33.33%, and the C/N ratio increased by 52.57% and 26.14%, respectively. In general, the effect of WSBC on improving soil carbon and nitrogen was superior to that of PSBC.

SOM is an important component of soil and an indicator of soil fertility. SOM and nutrients (N, P, K, etc.) have a direct interaction with soil fertility. Our results are consistent with those of previous studies in terms of the increase in SOM, AP, C, and N after the treatment of biochar [26,27]. It was not affected by the type of biochar or soil, and the change in AP might have been caused by a change in soil microbial activity.

### 3.3. Effect of Biochar on the Total Trace Elements in Soils

**Figure 2:**
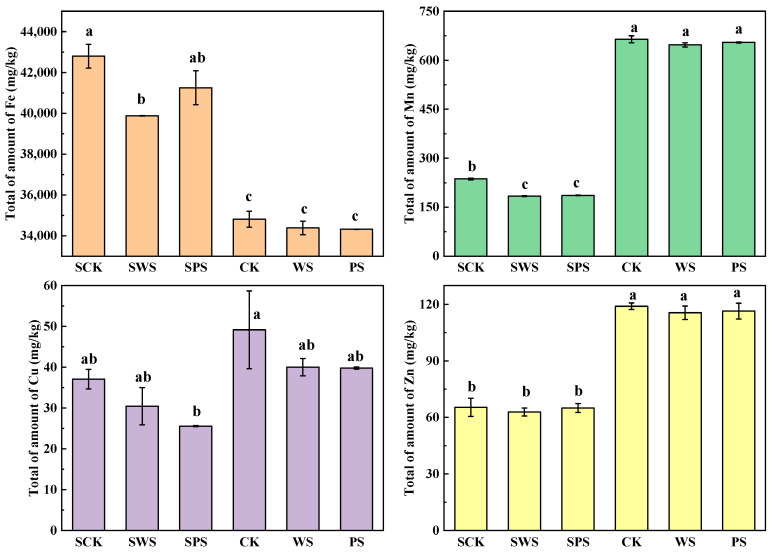
Effects of biochar on the total amounts of Fe, Mn, Cu, and Zn in the red soil and yellow-brown soil (error bars represent the standard deviation of the mean; different letters indicate significant differences (*p* < 0.05) among the treatment means).

The changes in the total contents of Fe, Mn, Cu, and Zn in the red and yellow-brown soils treated with biochar are shown in Figure 2. Specifically, the application of WSBC reduced the total content of Fe in the red and yellow-brown soils by 6.83% and 1.23%, respectively. Similarly, the total content of Fe also decreased by 3.26% and 1.42% after adding PSBC, and the difference was insignificant, especially in the yellow-brown soil. The total contents of Mn, Cu, and Zn in the red and yellow-brown soils also decreased with the application of biochar, and the application of WSBC reduced Mn, Cu, and Zn in the red and yellow-brown soils by 22.21%, 17.91%, and 3.83% and 2.55%, 18.60%, and 2.90%, respectively. The addition of PSBC also lowered them by 21.32%, 31.04%, and 0.58%, and 1.46%, 19.10%, and 2.17%, respectively. The decrease was significant only for Mn in the red soil (*p* < 0.05), and no significant changes were observed for Cu or Zn in either soil or for Mn in the yellow-brown soil.

The distribution of trace elements in the red and yellow-brown soils was significantly different in the order of Fe > Mn > Zn > Cu. The red soil exhibited a higher concentration of iron oxides and a more oxygen-rich composition compared to the yellow-brown soil, the content of Fe was 1.23 times higher than that in yellow-brown soil, while the contents of Mn, Cu, and Zn were lower than those in the yellow-brown soil. The addition of WSBC significantly reduced the Fe content in the red soil, while PSBC showed no significant effect on Fe content in either soil. No statistically significant reduction in Fe content was observed in the yellow-brown soil with either WSBC or PSBC. The observed decrease in Fe content in the red soil with WSBC may be attributed to potential mechanisms such as rice absorption or long-term irrigation leaching. The Fe in the yellow-brown soil with a higher pH was not significantly affected by the biochar, which may have been due to the adsorption of Fe by the biochar or the transformation of Fe minerals.

The total content of Mn in the yellow-brown soil was 2.81 times higher than that in the red soil. In the red soil, the content of Mn decreased by 22.21% after the addition of WSBC, which was probably due to the fact that the OH^−^ in the soil easily combined with the H^+^ obtained from the biochar, resulting in a decrease in H^+^ competitiveness and making more sites available for Mn adsorption [28]. The addition of biochar resulted in appreciable changes in the soil’s bulk density and porosity, thus changing the soil’s hydrological properties. There were also some differences in the soil’s water-holding properties after the addition of biochar. The differences in Mn content between SPS and SWS may have been partly due to the absorption of rice and partly due to the difference in water-holding properties due to the leaching out of irrigation water.

It was reported that the adsorption capacity of Cu and Zn increased with the decrease in the C/N ratio of biomass. The high adsorption capacity of biochar obtained from raw materials with a lower C/N ratio was expected due to the electrostatic attraction between the positively charged Cu and Zn ions and the delocalized π electrons on the aromatic structure of the biochar with larger specific surface areas [29]. As shown in Figure 2, the total content of Cu and Zn was reduced with the adsorption of WSBC and PSBC, but no significant difference was reduced for either soil or treatment.

The effect of biochar on the total amounts of Fe, Mn Cu, and Zn is influenced by the soil pH. In neutral soil, the effect of biochar on trace elements may be relatively small, as trace elements in neutral soil are usually in a relatively stable form. The pH of the yellow-brown soil was relatively small when biochar was added, so the effect of adding biochar on the total amounts of Fe, Mn Cu, and Zn in the yellow-brown soil was relatively small. Due to its high specific surface area and rich pore structure, biochar can adsorb trace elements in soil. Under acidic conditions, biochar has a better adsorption effect and is more likely to fix trace elements in soil, leading to a decrease in their total amount. After applying different biochar treatments to the red soil, there were significant differences in the total amounts of Fe and Mn. Specifically, the application of WSBC significantly reduced the total amount of Fe and Mn in the red soil, while the application of PSBC only significantly reduced the total amount of Mn; there was no significant difference in the total amount of Fe. There was no significant difference between the two biochar treatments.

### 3.4. Effect on the Availability of Trace Elements

**Figure 3:**
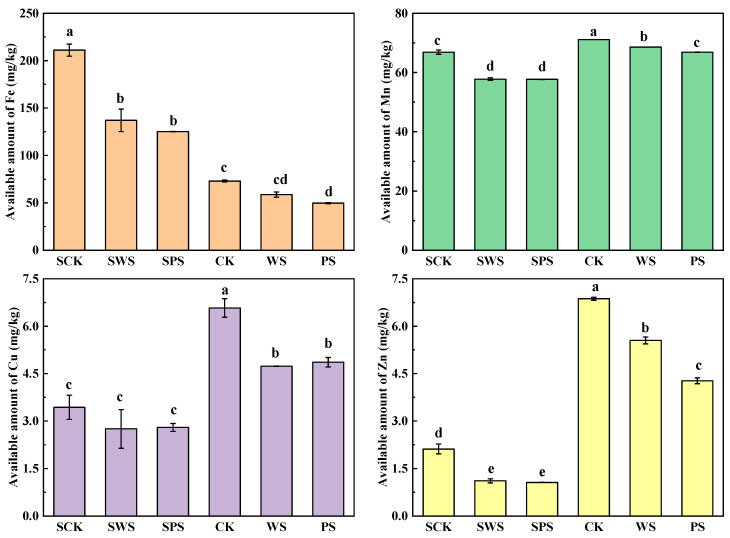
Effects of biochar on the availability of Fe, Mn, Cu, and Zn in the red soil and yellow-brown soil (error bars represent the standard deviation of the mean; different letters indicate significant differences (*p* < 0.05) among the treatment means).

Figure 3 shows the effects of WSBC and PSBC on the availability of Fe, Mn, Cu, and Zn in the red and yellow-brown soils. In the red soil, the application of WSBC and PSBC significantly decreased the available Fe, Mn, and Zn concentrations by 35.07%, 13.65%, and 47.31% and 40.67%, 13.73%, and 49.47%, respectively, whereas there were no significant differences between the two types of biochar. No significant differences were observed between the control red soil and the WSBC- or PSBC-treated soils in terms of the concentrations of available Cu. In the yellow-brown soil, the application of WSBC and PSBC showed significantly decreasing amounts of available Mn, Cu, and Zn compared with the control. Compared with the control, the content of available Fe and Cu decreased by 19.71% and 27.99% with the application of WSBC; this decrease was 31.87% and 26.08% with the application of PSBC, but there were no significant differences between the two types of biochar. The application of WSBC and PSBC decreased the available Mn and Zn by 3.59% and 19.17% and by 6.00% and 37.82%, respectively. There were significant differences between the two biochar treatments.

There was a significant difference in the available trace elements between the red and yellow-brown soils. The content of available trace elements in the yellow-brown soil was greater than that in the red soil, except for Fe, suggesting that the availability of trace elements in the yellow-brown soil was greater than that in the red soil. Since Fe and Mn (hydro)oxides are important reactive adsorption surfaces for trace elements, their release from the solid phase to pore water can impact trace elements’ leaching characteristics. Comparing the total amount and the available state of trace elements (Figure 2 and Figure 3), the addition of biochar significantly reduced the total amount of Mn and the available state of Fe and Mn in the red soil, in which WSBC significantly reduced the total amount of Fe, while the application of PSBC had no significant difference and the total Fe difference between the two biochar treatments was not significant. In the yellow-brown soil, the addition of biochar had no significant effect on the total amount of Fe and Mn, among which WSBC significantly reduced the content of available Mn and PSBC had a significant effect on the available state of bot Fe and Mn. The application of biochar can enhance microbial activities, thus accelerating O_2_ consumption and, thereby, inducing the onset of reducing conditions [30]. Fe and Mn release is susceptible to changes in redox, and a reduction in Fe and Mn can improve their solubility. Soil contains much more Fe than Mn, while Mn (II) is more stable in anoxic water than Fe (II); additionally, the addition of biochar improves the flow of soil pore water, so there was no significant difference between the two types of biochar in terms of the concentration of available Mn in the soil.

The effects of WSBC and PSBC on Cu and Zn in the red soil were consistent. The addition of biochar significantly reduced the content of available Zn in the red soil and the available state of Cu and Zn in the yellow-brown soil, but it had no significant effect on the total amount of Cu and Zn in both soils. After adding WSBC and PSBC to the acidic red soil, the soil pH and organic matter content improved, making it more suitable for the growth of rice. Therefore, the availability of Cu and Zn in the red soil was reduced, but there were no significant differences. Since the pH of WSBC was greater than that of PSBC, the Cu in the yellow-brown soil was fixed by WSBC, and its availability was reduced. However, the synergistic effects of the alkalinity and ash of the biochar in the PSBC treatment were not as large as those in the WSBC treatment. Therefore, the availability of Cu in the yellow-brown soil treated with PSBC was higher than that in the WSBC treatment, but the difference between the two biochar treatments was not significant. Zn was very mobile in the soil environment, especially in low-pH conditions. At a relatively lower pH, the oxygen-containing functional groups in biochar play a crucial role in the complexation of Zn, while, at a higher pH value, silicate plays a leading role in the adsorption of Zn [31]. Hence, the availability of Zn was reduced after adding WSBC and PSBC to the acidic red soil, and the decrease was greater than that in the yellow-brown soil, but the difference between the two biochar treatments was not significant. After adding biochar to the yellow-brown soil, the availability of Zn was significantly higher in the treatment with WSBC than in the treatment with PSBC. This might be due to the competition of Fe, Mn, and Cu for carbonate being greater than that of Zn, thus increasing the availability of Zn. The availability of Zn was significantly reduced by the addition of PSBC, which might have been because of the interaction between bacteria and organic components in the soil [32].

### 3.5. Effect of Biochar on Trace Elements in Rice Grains

**Figure 4:**
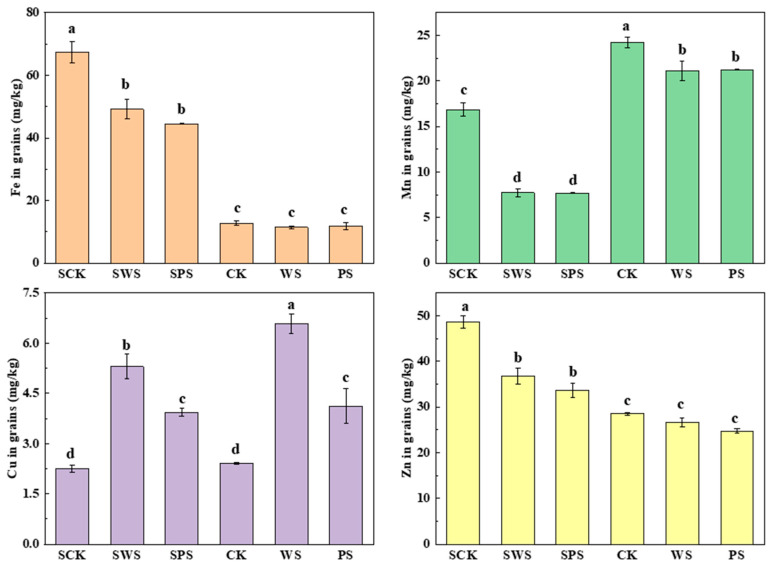
Effects of biochar on the Fe, Mn, Cu, and Zn concentrations in rice grains (error bars represent the standard deviation of the mean; different letters indicate significant differences (*p* < 0.05) among the treatment means).

The effects of biochar on the Fe, Mn, Cu, and Zn concentrations in rice grains in the red and yellow-brown soils treated with biochar are shown in Figure 4. The application of WSBC reduced the content of Fe in rice grains in the red soil and yellow-brown soil by 27.06% and 10.81%, respectively. Similarly, the content of Fe in rice grains also decreased by 33.89% and 7.43% after adding PSBC, and there were no significant differences between the different types of biochar, especially in the yellow-brown soil. With the application of biochar, the contents of Mn and Zn in rice grains in the red soil and Mn in rice grains in the yellow-brown soil decreased. WSBC reduced the contents of Mn and Zn in rice grains in the red and yellow-brown soils by 54.11% and 24.58% and by 13.04% and 6.47%, respectively. The addition of PSBC also decreased these contents by 54.44% and 30.78% and by 12.37% and 13.19%, respectively. However, the content of Cu in rice grains increased significantly; the Cu content in grains in the red soil and yellow-brown soil increased by 134.90% and 171.91 after adding WSBC, respectively, and the addition of PSBC increased the content by 74.59% and 70.54%, respectively.

In this study, there was a significant difference in rice grains’ trace elements between red and yellow-brown soils. The contents of the trace elements of rice grains were in the following order: Fe > Zn > Mn > Cu. The contents of Fe and Zn in rice grains in the red soil were significantly higher than those in the yellow-brown soil, while the contents of Mn and Cu were lower than those in the yellow-brown soil. The application of biochar reduced the contents of Fe, Mn and Zn in the red soil and Mn in the yellow-brown soil in grains, which demonstrates that the application of biochar decreased the enrichment of Fe, Mn, and Zn in the corresponding soil in the grains. The content of Cu in grains increased significantly, which may have been due to the decrease in the soil availability of Cu caused by biochar and the influence of rice growth. Previous studies also reported that biochar was utilized to enhance micronutrient concentrations, such as those of Zn and Cu, in cereal and vegetable crops [33,34]. Gonzaga et al. [35] reported that, despite the reduction in soil Cu availability due to biochar application, it did not manage to reduce Cu uptake by Indian mustard, which was probably due to the mustard’s high absorption efficacy.

### 3.6. Relationship Between Soil’s Physical and Chemical Properties and Trace Element Content and Bioavailability

In the wake of continuous climate change, the demand for high-quality rice grains has become increasingly evident [36]. The improvement of mineral nutrition in rice grains includes the selection of rice genotypes with a greater accumulation of essential minerals (i.e., Fe, Mn, Zn, etc.) [37]. Sohi et al. [38] found that the nutrient concentration in plants depends on soil quality, nutrient availability, and biochar characteristics. However, the correlation between nutrients in soil and plants also depends on the interactions among nutrients in soil and possible synergisms/antagonisms, affecting plant uptake [39]. Biochar application is reported to increase nutrient bioavailability in soils [40] and to positively affect grain yield [41,42,43].

**Figure 5:**
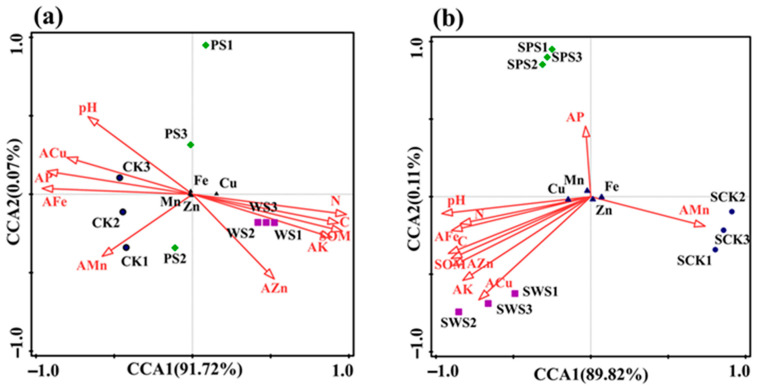
A Canonical correlation analysis (CCA) between trace elements and environmental factors in grains ((**a**): red soil; (**b**): yellow-brown soil; the red arrows represent various environmental parameters).

In order to study the correlation between trace elements in rice grains and the availability of trace elements in the soil, some environmental factors were selected for a canonical correlation analysis (CCA). According to the results of the CCA (Figure 5a), Fe, Mn, and Zn in grains are negatively correlated with the available state in the soil, while Cu is positively correlated with the available state in the soil. In Figure 5b, it can be seen that there is a positive correlation between Fe, Mn, and Zn in grains and the corresponding available state in soil, while Cu is negatively correlated with the corresponding available state in soil. Fe, Mn, Cu, and Zn are inversely proportional to the corresponding available content in different soils, as the remaining available content in the soil decreases after being absorbed by plants, which is consistent with our hypothesis. Fe, Mn, Cu, and Zn show the opposite trends in the two soils, which may be related to the properties of the soil.

After biochar was added to the red soil, the Fe in grains was negatively correlated with pH, C, N, SOM, available phosphorus, and available potassium, while Mn in grains was the opposite to Fe in grains, indicating that there was a competitive relationship between Fe and Mn in the grains; additionally, Cu and Zn in grains were negatively correlated with available phosphorus. When biochar was added to the yellow-brown soil, Fe, Mn, and Zn were positively correlated with soil pH and available phosphorus, while Cu was negatively correlated with pH and available phosphorus. In conclusion, trace elements in grains are closely related to soil physical and chemical properties, especially available phosphorus. Mn in rice grains is positively correlated with available phosphorus, while Cu is negatively correlated with available phosphorus, demonstrating that there is a competitive relationship between Mn and Cu in the grains. Fe and Zn in rice grains are negatively correlated with available phosphorus in the red soil, while Fe and Zn are positively correlated with available phosphorus in the yellow-brown soil. This could prove that Fe and Zn in grains are influenced by available phosphorus in the soil. Therefore, it is necessary to further study whether the relationship between Fe and Zn in grains and available phosphorus in soil are related to the soil type and biochar addition.

## 4. Conclusions

WSBC and PSBC were applied to red soil and yellow-brown soil to carry out rice pot experiments, and we found that the improvement in soil nutrients is not limited by the type of biochar. The increasing effect of WSBC on SOM, AK, C, and N was better than that of PSBC, and the soil AP was affected by the types of biochar. In the red soil, the content of Fe and Mn in total, available and grains decreased after the addition of WSBC, while the contents of total Mn, available and grains Fe, Mn and Zn decreased after the addition of PSBC. In the yellow-brown soil, the content of available Mn, Cu, Zn and Mn in grains decreased after the addition of biochar. The content of Cu in the grains of the two soil treatments was increased, which was related to the adsorption of biochar, plant absorption, interaction between trace elements, and irrigation leaching during the growth of rice. The content of available Mn, and Zn in the yellow-brown soil treated with WSBC was higher than that of samples treated with PSBC, and the contents of Cu in the grains treated by WSBC in both soils were higher than those in the PSBC treatment. The content of total and available Cu and Zn in the yellow-brown soil was higher than that in the red soil, but the content of Zn in grains was lower than that in the red soil, which was related to the nature of the soil. CCA showed that Fe, Mn, and Zn in red soil and grains and Cu and Zn in yellow-brown soil were negatively correlated with the corresponding soil’s available state, which was consistent with our hypothesis. There was a significant positive correlation between Cu content in the grains and the availability of Cu content in the red soil, which may have been related to the competition between Cu and Zn in grains. Fe and Mn in yellow-brown soil grains are positively correlated with the corresponding available content, which may be related to the occurrence form of trace elements in the soil, thus affecting the content in grains. Accordingly, WSBC can be applied to soil to enhance soil nutrients, and PSBC can be applied to reduce the availability of Fe, Mn and Zn, especially in acidic soils, where biochar is more effective.

## Figures and Tables

**Table 1:** Basic physical and chemical properties of pH, TC, TN, C/N, available phosphorus (AP), available potassium (AK), soil organic matter (SOM), and the total amount of trace elements in red and yellow-brown soils.

Type	pH	C/%	N/%	C/N	AP/ mg/kg	AK/ mg/kg	SOM/ g/kg	Fe/ g/kg	Mn/ mg/kg	Cu/ mg/kg	Zn/ mg/kg
Red soil	4.92 ±0.01	4.23 ±0.15	0.367 ±0.12	13.67 ±0.00	0.580 ±0.23	21 ±0.78	4.09 ±0.32	39.99 ±0.14	199.8 ±4.05	20.56 ±2.16	55.65 ±0.95
Yellow-brown soil	7.48 ±0.02	31.2 ±0.12	280 ±0.01	11.14 ±0.36	56.6 ±3.13	118 ±1.75	8.64 ±0.22	37.80 ±3.41	738.5 ±6.49	34.73 ±1.80	157.9 ±1.48

Note: Data in the table represent means ± standard deviations.

**Table 2:** Physical and chemical characteristics of pH, ash, CEC, C, N, C, total nutrient (N, P, K), SBET, and the total amount of trace elements in WSBC and PSBC.

Type	pH	Ash/ %	CEC/ cmol/kg	C/ %	N/ %	N/ g/kg	P/ g/kg	K/ g/kg	SBET /m^2^/g	Fe/ mg/kg	Mn/ mg/kg	Cu/ mg/kg	Zn/ mg/kg
WSBC	11.87 ±0.01	28.99 ±1.39	38.71 ±2.11	64.5 ±0.02	0.810 ±1.94	10.24 ±1.31	0.460 ±0.04	15 ±0.96	4.58 ±0.00	1.619 ±0.13	0.4215 ±0.02	0.0007 ±0.00	0.0232 ±0.00
PSBC	11.44 ±0.02	39.67 ±3.36	23.77 ±2.00	56.1 ±0.02	1.34 ±0.61	10.55 ±1.66	0.990 ±0.05	2.9 ±0.00	3.52 ±0.00	2.986 ±0.12	0.3858 ±0.01	0.0198 ±0.00	0.3516 ±0.01

Note: Data in the table represent means ± standard deviations.

**Table 3:** Soil pH, SOM, AP, AK, C, N, and C/N ratio after WSBC and PSBC treatments in red soil and yellow-brown soil.

Treatment	pH	SOM/g/kg	AP/mg/kg	AK/mg/kg	C/g/kg	N/g/kg	C/N
SCK	5.21 ± 0.01 e	6.62 ± 0.21 e	0.597 ± 0.09 c	91 ± 1.51 f	3.65 ± 0.21 f	0.465 ± 0.05 c	7.92 ± 1.30 d
SWS	6.40 ± 0.04 c	51.23 ± 0.37 b	1.69 ± 0.19 c	1151 ± 6.10 a	27.9 ± 0.06 c	0.660 ± 0.07 c	42.49 ± 5.52 a
SPS	6.13 ± 0.08 d	25.09 ± 0.63 d	1.23 ± 0.06 c	514 ± 9.02 c	13.4 ± 0.06 e	0.575 ± 0.05 c	23.44 ± 3.01 b
CK	7.70 ± 0.01 a	40.73 ± 0.65 c	47.4 ± 0.78 b	134 ± 3.42 e	24.5 ± 0.21 d	2.29 ± 0.02 b	10.70 ± 0.19 cd
WS	7.59 ± 0.04 b	77.28 ± 0.46 a	49.0 ± 0.52 b	875 ± 1.89 b	43.4 ± 0.42 a	2.67 ± 0.18 a	16.33 ± 1.24 c
PS	7.74 ± 0.02 a	49.81 ± 1.11 b	55.7 ± 1.56 a	396 ± 7.21 d	32.6 ± 0.57 b	2.42 ± 0.01 b	13.50 ± 0.20 cd

Note: Data in the table represent means ± standard deviations, different lowercase letters after the same column indicate significant differences among treatments (*p* < 0.05).

## Data Availability

No new data were created.
